# The efficacy of maternal health education and maternal screening on knowledge and the uptake of infant screening for sickle cell disease in Dar-Es-Salaam, Tanzania; a quasi experimental study

**DOI:** 10.1186/s12889-022-14859-2

**Published:** 2023-01-10

**Authors:** Hilda J. Tutuba, Agnes Jonathan, William Lloyd, Upendo Masamu, Emanuela Marco, Julie Makani, Paschal Ruggajo, Benson R. Kidenya, Irene K. Minja, Emmanuel Balandya

**Affiliations:** 1 Sickle Pan-African Research Consortium (SPARCO), Dar-Es-Salaam, Tanzania; 2 Department of Hematology and Blood Transfusion, Sickle Cell Program, MUHAS, Dar-Es-Salaam, Tanzania; 3 Department of Physiology, MUHAS, Dar-Es-Salaam, Tanzania; 4 Department of Internal Medicine, MUHAS, Dar-Es-Salaam, Tanzania; 5 Department of Biochemistry and Molecular Biology, Catholic University of Health and Allied Sciences- Bugando, Mwanza, Tanzania; 6 Department of Restorative Dentistry, MUHAS, Dar-Es-Salaam, Tanzania

**Keywords:** Sickle cell disease (SCD), Efficacy, Health education, Maternal screening for SCD, Intervention, Knowledge, Infant screening

## Abstract

**Background:**

Globally, Sickle cell disease (SCD) is one of the most common genetic disease with high childhood mortality. Early identification of babies with SCD through newborn screening (NBS) and linking them to care are among the recommended interventions. The purpose of this study was to assess the efficacy of maternal health education and maternal screening for SCD on knowledge and the uptake of infant screening for SCD among mother-infant pairs attending antenatal clinics at Government health facilities in Dar-es-salaam, Tanzania.

**Methods:**

This study was a pre-test post-test, quasi-experimental which involved pregnant women attending antenatal clinics at three hospitals; Mbagala hospital, Sinza hospital and Buguruni health center in Dar Es Salaam. A structured questionnaire was used in data collection. Knowledge on SCD was assessed for all participants before and after two sessions of health education. Participants in Mbagala and Buguruni were also screened for SCD using Sickle SCAN point-of-care test (BioMedomics Inc, USA). The efficacy for health education intervention was computed as the post-intervention minus baseline knowledge score. For proportions, a two-sample z-test was used. Univariate and multivariate logistic regression were used to analyze the efficacy of health education intervention and also predictors of infant diagnosis.

**Results:**

For two sessions of health education intervention, a total of 467 pregnant women completed the sessions. During antenatal visits, a total of 218 were screened for SCD. The proportion of participants with good knowledge of SCD had significantly increased to 85.9% from 12.4% at baseline following the education intervention. In multivariate analysis, sharing the received education on SCD was an independent predictor of the efficacy of health education intervention. Maternal occupation, maternal SCD status as well as sharing the received education on SCD were independent predictors of the uptake of SCD infant diagnosis.

**Conclusion:**

This study has demonstrated that maternal health education and maternal screening for SCD are feasible and efficacious interventions in raising knowledge and improving the uptake of infant diagnosis for SCD. These interventions are strongly recommended to be included in the comprehensive care package for pregnant women attending antenatal clinics, particularly in areas with a high burden of SCD.

## Introduction

Sickle cell disease (SCD), a genetic disorder of the globin chain of the red blood cells, is one of the greatest public health problems of this age [1]. SCD affects millions of people worldwide and is more common among those whose ancestors came from sub-Saharan Africa. It is estimated that 300,000 – 500,000 babies are born every year with SCD worldwide, with estimation of 75% being from Africa [1]. In Tanzania, 8,000 – 11,000 infants with SCD are born annually compared to 1,000 infants in USA [1, 2] with estimated sickle cell trait of between 13 to 20% [3, 4].

Until the 1970s, the management of SCD was passive, which led to poor outcomes among SCD patients. In the present age, there is significant improvement in clinical outcomes and patient survival at large, which are due to the availability of improved SCD services such as newborn screening services, vaccination, antibiotic prophylaxis, health education programs to patients and caregivers as well as early identification and treatment of complications, which are provided since birth [1, 5].

Management of SCD has improved significantly, especially in developed countries [1]. In middle- and lower-income countries, where the burden of SCD is high, the management of SCD is still suboptimal including lack of awareness and proper knowledge, inadequate screening, diagnosis and treatment [6]. In these settings, SCD diagnosis is often made for the first time when there are already complications due to the disease. Early diagnosis and management will help to reduce complications, morbidity and mortality due to SCD [1, 6, 7].

Newborn screening allows for the early diagnosis of newborns with SCD and their linkage to close medical follow-up [8]. The World Health Organization has recommended newborn screening as a key strategy for reducing pediatric mortality in Africa, where infants with SCD face an estimated 50–90% early childhood mortality, following success of universal newborn SCD screening in many developed countries that resulted in significant decrease of pediatric SCD mortality [9]. Unfortunately, most countries in sub-Saharan Africa, including Tanzania, do not have universal newborn screening programs for SCD. Further, community health education platforms, such as antenatal clinics (ANC), are largely underutilized [10]. In Tanzania for instance, while up to 94% of pregnant women reported to make at least one ANC visit before delivery, it is only 47% who reported to have been informed of the danger signs in pregnancy [10], and health education on SCD is not routinely provided.

The efficacy of health education intervention on knowledge and the uptake of infant screening for SCD has not been studied in Tanzania. Further, it is not known whether empowering mothers with the knowledge of personal SCD status will enhance the uptake of infant diagnosis for SCD. We conducted this study to assess the efficacy of maternal health education and maternal screening for SCD on knowledge and the uptake of infant screening for SCD among mother-infant pairs attending antenatal clinics at Government health facilities in Dar-Es-Salaam, Tanzania. The results from this study will be useful to policy makers and health care providers in considering integrating health education on SCD, maternal screening and infant screening for SCD on available antenatal care package and Reproductive and Child Health (RCH).

## Materials and methods

### Study design, study population and study setting

This was a pre-test post-test, quasi-experimental study conducted at three sites (Mbgala, Buguruni and Sinza).

In this study, we assessed two interventions, which were:

- **Health education intervention on knowledge**: By using one group pre-post-test design where all study participants controlled for themselves by being assessed before health education intervention and after intervention.

- **Maternal screening for SCD intervention**: Where all participants from Mbagala and Buguruni sites received the intervention (screened for SCD) while those from Sinza did not receive the intervention (not screened), hence served as the control group for the screening intervention.

This study involved all pregnant women who were attending the antenatal clinics during our study period (2020 to 2022). We had three study sites; Mbagala hospital, Buguruni health center and Sinza hospital, which are all primary level public health facilities, providing services to urban population in the City of Dar Es Salaam. There is no dedicated SCD clinics from either of the sites, but all are close to the regional referral hospitals which run SCD clinics. Currently, Tanzania does not have a universal newborn or infant screening program for SCD. Services are available at some health facilities, including the regional referral hospitals and national hospital located in Dar-es-salaam. Participants were directed to attend to these facilities upon delivery for their babies to be screened for SCD.

### Sample size and sampling technique

The sample size of 440 participants was obtained from the formula,

$${\varvec{m}}1=\frac{{\left[{{\varvec{z}}}_{{}^{{\varvec{a}}}\!\left/ \!{}_{2}\right.}\sqrt{\left({\varvec{r}}+1\right){\varvec{p}}\left(1-{\varvec{p}}\right)}+{{\varvec{z}}}_{{\varvec{\beta}}}\sqrt{{{\varvec{r}}{\varvec{p}}}_{0}+\left(1-{{\varvec{p}}}_{0}\right)+{{\varvec{p}}}_{1}\left(1-{{\varvec{p}}}_{1}\right)}\right]}^{2}}{{\varvec{r}}{\left({{\varvec{p}}}_{0}-{{\varvec{p}}}_{1}\right)}^{2}}$$

where; ${\varvec{p}}=\frac{{{\varvec{p}}}_{0}+{{\varvec{r}}{\varvec{p}}}_{1}}{{\varvec{r}}+1}$, P^0^ is the proportion in population receiving HE only, and P^1^ is the proportion in population receiving HE and maternal screening, r is the case and control ratio.

Assumptions: alpha = 0.05 (two sided), Power = 95%, P^0^ = 0.5, P.^1^ = 0.35, **m0/m1** = *r* = 1

$$Attrition rate = 20\%$$

$${\varvec{M}}1\boldsymbol{ }=\boldsymbol{ }220$$

The minimum required sample size for this study was 440 participants, being 220 to be screened and receive health education and 220 to receive health education only without screening. Additional 160 participants were recruited to get the final total of 600 participants who were enrolled into the study. Convenient sampling technique was used to select the participants where all pregnant women available at our study sites during the study period, who were willing to take part in our study and met the inclusion criteria were selected and enrolled in our study. The inclusion criteria were; pregnancy at gestation age between 20–28 weeks during first visit (since those with gestation age above 28 weeks during first visit were likely to deliver before completing the series of health education interventions planned in the study); absence of pregnancy complications which risk termination of pregnancy (such as heart failure, pregnancy-induced hypertension, threatened abortion, hyperemesis gravidarum, gestational diabetes), and not having received blood transfusion within the past four months prior enrolment into the study as the donor blood could interfere with the results of SCD screening test (this last criterion was considered only among participants in the screening arm).

Health education intervention was provided to all participants from each of the three sites (Buguruni, Mbagala and Sinza health facilities) where each participant controlled for herself using the pre-post-test design while on the screening intervention, participants from Buguruni and Mbagala sites served as intervention group (were screened for SCD) while participants from Sinza served as control group (not screened for SCD). This separation of maternal screening by sites was done to ensure that pregnant women at any particular site received same interventions, thus mitigate concerns regarding preferential treatment and also prevent mix-up of interventions across participants. The three study sites were selected purposively based on their proximity to the referral hospitals with available SCD services where participants could be referred to.

### Enrollment, data collection and follow-up

This study had three phases;

1. Pre-intervention phase (1 day)

Eligible participants provided informed consent prior enrolment into the study. Following informed consent, a structured questionnaire was administered by a research assistant to each participant. The questionnaire gathered demographic information and contained questions assessing the baseline level of knowledge on SCD (if SCD is related to blood cancer, life expectance of patients SCD, knowledge on the mode of acquiring and diagnosis of SCD as well as the most common signs and symptoms of SCD).

(2) Intervention phase (2 months)

After administration of the questionnaire, the first session of health education on SCD was conducted physically at the study sites to either one participant or to a group with maximum number of seven participants, depending on their availability. We used the health education materials which are available at Muhimbili University of Health and Allied Sciences—Sickle Cell Program and are being used in proving health education to the community. These materials covered what SCD is, mode of acquiring and diagnosis, signs and symptoms, complications and cleared different myth on SCD. Each health education session lasted in average of 15 min. However, we provided room for questions and answered all concerns raised by the participants regarding SCD. Further, proper information was given to participants on where and how to access the extended services for SCD involving SCD clinics, infant screening services and diagnostic services. We also provided to study participants contact information for enquiry in case someone wanted to get additional clarification on SCD.

Participants from the screening sites were freely screened for SCD using the Sickle SCAN® point-of-care test following manufacturer’s instructions (BioMedomics Inc., United States). The test is capable of identifying Hemoglobin A, S and C variants in blood samples. We conducted maternal screening for SCD at antenatal laboratories where other routine screening activities such as HIV and hemoglobin level took place. Each participant was provided with results of her SCD test on-site. Counselling was given and information was provided on where to get additional SCD services.

In one to two months after the first health education session, the second session of health education was conducted upon follow-up of all 600 participants through phone call where every participant was called in-person. All participants who were reachable and were willing to continue with the study received the education by using the same health education materials used in the first session.

(3) Post intervention phase (15 months)

**Fig. 1:**
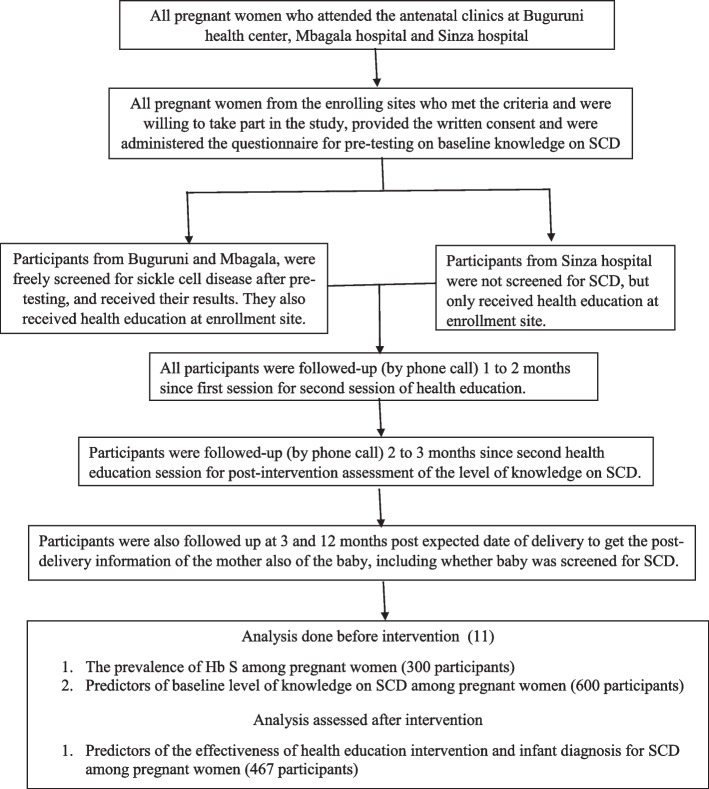
The Flow chart of participants involved in the study from enrollment to follow-up

Two to three months after the second health education session, the post-intervention assessment of the level of knowledge on SCD was conducted to all participants using the same questionnaire that was used for pre-testing. During the education sessions, participants were informed of the locations at the regional referral hospitals and national hospital where SCD screening services can be obtained for their babies upon delivery. Three months after the respective expected dates of delivery (which were collected at enrollment), the post-delivery information of both mother and the baby was collected, including whether the baby was screened for SCD. The latter information was collected for the last time twelve months post expected date of delivery (Fig. 1). All participants who could not be reached three times in different days, those who were not willing to continue with the study as well as the death of the participant, were termed as loss to follow-up.

### Statistical analysis

In this study, we had two dependent variables which were: Maternal level of knowledge on SCD and the uptake of infant screening for SCD after delivery. The independent variables were: Age, Level of education, Occupation, Marital status, Maternal screening for SCD, Maternal SCD status, Remembering personal SCD Status and Sharing health education received.

We used Stata Version 17 for data analysis. We expressed the descriptive statistics such as the socio-demographic characteristics of the participants and their babies in frequencies and percentages, and presented in tables.

We graded the level of knowledge depending on the number of correct responses scored from the questionnaires which was constructed from different literature that assessed the level of knowledge on SCD in different groups including pregnant women, also modified to fit the local context [11–13]. A correct response was scored as “1” while a wrong response was scored as “0”. Total knowledge score ranging from 0 to 10 was calculated as the sum of all correct responses considering the following score range; < 7 poor knowledge, 7–10 good knowledge. We used the two-sample Z test for proportions to ascertain statistical significance of the difference in proportions of the participants with good knowledge on SCD before and after health education intervention. Chi-square test was used in ascertaining the efficacy of maternal screening on the uptake of infant screening for SCD between sites where maternal screening for SCD was provided and those where maternal screening was not provided. Further, we computed “*efficacy*” of health education intervention as the post-intervention minus baseline score for each individual participant, where optimal efficacious was considered to have been achieved in those who gained 3 points or higher. Subsequently, we dichotomized participants into those where health education intervention was “efficacious” versus those where intervention was “not efficacious” and performed univariate as well as multivariate logistic regression to ascertain the predictors of the efficacy of health education intervention. Further, we performed inferential statistics on predictors of infant screening by comparing the different categories between the dependent variable (if child screened for SCD) with various independent variables (maternal level of education, occupation, maternal screening for SCD, maternal SCD status, level of knowledge post health education intervention and if she shared the health education received) using regression analysis where all independent variables associating with infant diagnosis with p-values of 0.2 or less during univariate logistic regression were included in the final multivariable logistic regression model. We considered a two tailed *p*-value below 0.05 to be statistically significant.

## Results

### Enrolment, interventions, and follow-up

**Fig. 2:**
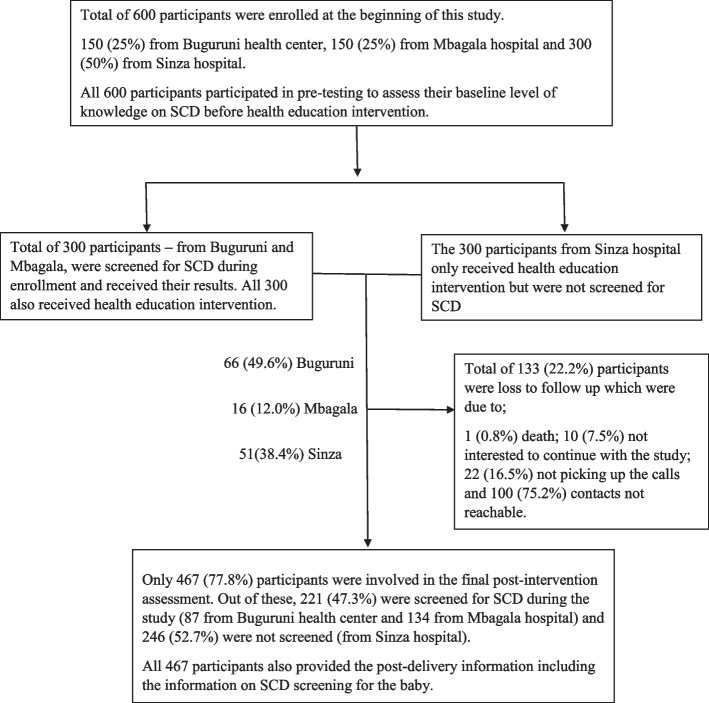
The Flow chart of participants involved in the study from enrollment to follow-up

Baseline level of knowledge on SCD was assessed on all 600 participants. Half of the participants (300) were screened for SCD (25% from Buguruni health center and 25% from Mbagala hospital) and the other half, 300, were not screened (from Sinza hospital). All 600 participants (100%) received the first session of the health education intervention, 477 (79.5%) received the second session and 467 (77.8%) were available for post-intervention assessment of the level of knowledge on SCD and provided information on the status of SCD screening for their babies (Fig. 2).

In total, 133 (22.2%) participants were loss to follow up where 82 (13.7%) were from the screened arm (11.0% from Buguruni health center and 2.7% from Mbagala hospital). Of the 133 loss to follow-up participants, 10 (7.5%) were not interested to continue with the study, 22 participants (16.5%) did not pick up their phone calls and the phone contacts of 100 participants (75%) were not reachable (Fig. 2).

### Socio-demographic characteristics of the pregnant women enrolled into the study

Out of 467 participants who were involved in this study, most of them were of the age between 26—35 years old 232 (49.7%). Majority were married 391 (83.7%), with secondary level of education 206 (44.1%) and self-employed 271 (58.0%). Most of the participants 345 (73.9%) visited to antenatal clinics five times or more before delivery and 371 (79.4%) of the participants delivered at the place of enrollment (Table 1).

**Table 1:** Socio-demographic characteristics of pregnant women enrolled into the study (*n* = 467)

Maternal characteristics	N	Percent (%)
**Age (years)**		
< 18	1	0.2
18 – 25	165	35.3
26 – 35	232	49.7
> 35	69	14.8
**Marital status**		
Married	391	83.7
Not married	76	16.3
**Level of education**		
Illiterate	10	2.1
Primary level	188	40.3
Secondary level	206	44.1
College / university	63	13.5
**Occupation**		
Employed	47	10.1
Self employed	271	58.0
House wife	149	31.9
**Health Facility visited during antenatal**		
Buguruni Health Center	84	18.0
Sinza Hospital	249	53.3
Mbagala Hospital	134	28.7
**Number of antenatal visits**		
One visit	0	0
Two visits	2	0.4
Three visits	14	3.0
Four visits	206	22.7
Five or more visits	345	73.9
**Place of delivery**		
Place of enrollment	371	79.4
Other primary health facilities	53	11.3
Regional or referral hospital	43	9.2
**Maternal screening for SCD**		
Yes	221	47.3
No	246	52.7
**Place of Screening (*n* = 221)**		
During enrollment	220	99.5
Other primary health facilities	0	0
Regional or Referral hospitals	1	0.5
**Maternal SCD Status (*n* = 221)**		
AA	190	85.9
AS	30	13.6
SS	1	0.5
Other types	0	0
**If remembers Personal Status (*n* = 221)**		
Yes	203	91.9
No	18	8.1
**If Shared received health education(*n* = 467)**		
Yes	309	66.2
No	158	33.8
**Relation to those shared (*n* = 309)**		
Partner / Children	223	72.2
Parents / Siblings	29	9.4
Friends and others	57	18.4
**The outcome of sharing health education with others (*n* = 309)**		
Encouraged me to screen / screen my baby	18	5.8
Motivated others to get more information on SCD	227	73.5
Motivated others to screen	7	2.3
They ignored the information given	57	18.4

Of the 467 participants 221 (47.3%) who were screened for SCD. Majority of them had genotype of hemoglobin AA 190 (85.9%) and 1(0.5%) participant had hemoglobin SS, who had never screened before and did not know of her sickle cell status. Out of 221 participants who were screened, 203 (91.9%) remembered their sickle cell status 3 to 4 months after screening (Table 1).

A total of 467 participants received two sessions of health education where 309 (66.2%) of them shared the received education with others. Among those who shared the received education, 223 (72.2%) shared with their partners and children and majority of them motivated others to get more information on SCD 227 (73.5%), while 7 (2.8%) motivated others to screen for SCD (Table 1).

### Socio-demographic characteristics of the babies born to pregnant women enrolled into the study

Total of 470 babies were born to 467 mothers who were involved in this study where 3 mothers had twins. Of the 470 babies, 250 (53.2%) were female. Most of the babies 445 (96.3%) paid a total of three RCH visits for vaccination – which was the right number of visits required at their age, while only 26 (5.5%) were screened for SCD. Following the results of those screened, 23 (88.5%) babies had the genotype of hemoglobin AA, while the 3 (11.5%) babies had hemoglobin SS, two of them being of the mother who was diagnosed to have HbSS during this study, and all of them have been enrolled in SCD clinics. Mothers whose babies were not screened for SCD had different reasons for not screening them, most of it being forgetting to take their babies to health facilities for screening (Table 2).

**Table 2:** Socio-demographic characteristics of babies born to pregnant women enrolled into the study

Babies characteristics	N	Percent (%)
**Sex (*n* = 470)**^a^		
Male	240	46.8
Female	250	53.2
**Birth weight (*n* = 470)**		
< 2.5	14	3.0
2.5 to 3.9	436	92.8
4 and above	20	4.2
**Attendance to RCH clinic within the first three months of life (*n* = 470)**		
0	8^b^	1.7
1	0	0
2	17	3.6
3	445	94.7
**Child screened for SCD (*n* = 470)**		
Yes	26	5.5
No	444	94.5
**Reason for not screening (*n* = 444)**		
Financial challenges	106	23.9
Mather forgot about it	237	53.4
Sickness of either mother or child	16	3.5
Thought not important	42	9.5
Death of the child	8	1.8
Up region/no screening equipment	24	5.4
Other reasons	11	2.5
**Age (months) Screened (*n* = 26)**		
0 to 3	6	23.2
4 to 6	3	11.5
7 to 9	5	19.2
10 to 12	5	19.2
13 to 16	7	26.9
**Child Status (*n* = 26)**		
AA	23	88.5
AS	0	0
SS	3	11.5

^a^Total number of 470 children were born from 467 mothers where 3 babies were twins

^b^Total of 8 children died before their first visit to RCH clinic

### Responses to questions on knowledge on SCD before and after health education intervention

**Fig. 3:**
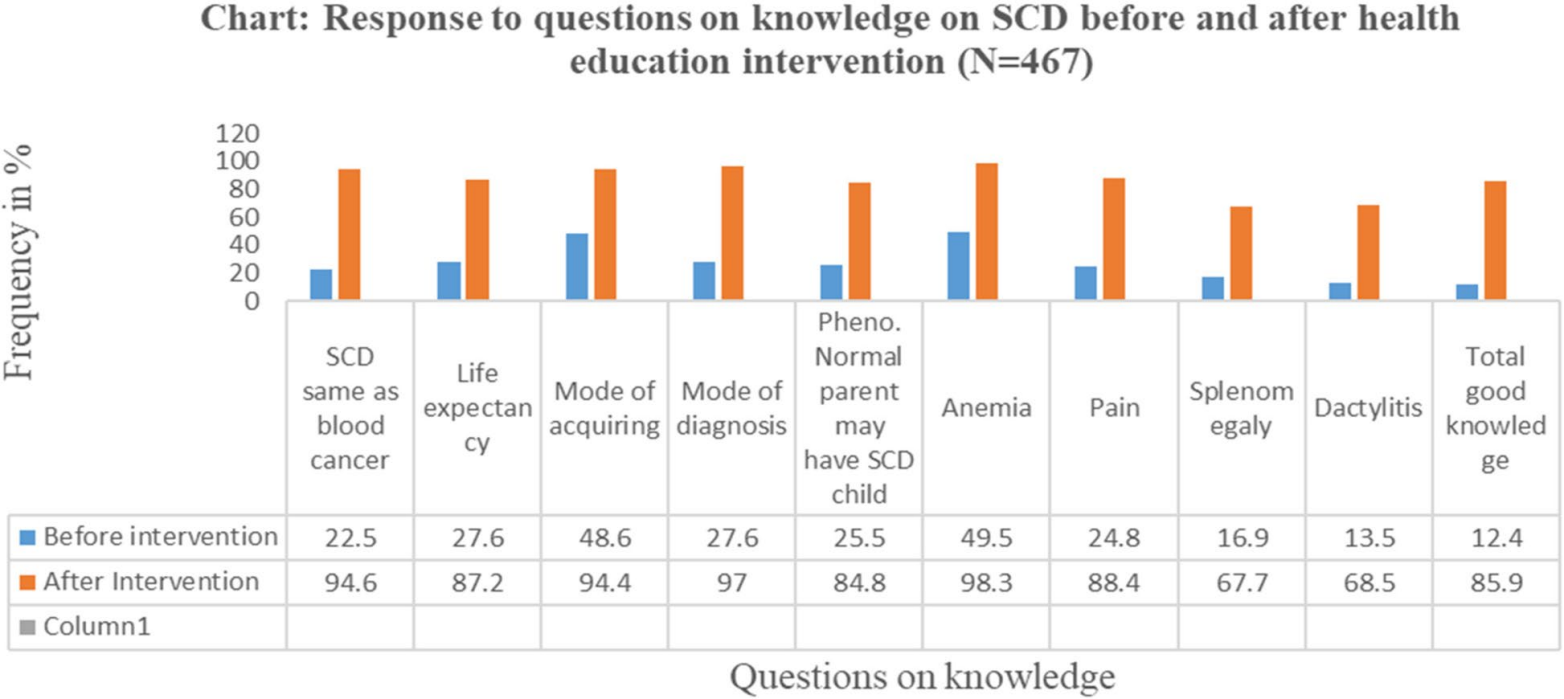
Response to Questions on Knowledge on SCD before and after health education intervention (*N* = 467)

The proportion of participants with good overall knowledge on SCD had significantly increased from 12.4% [9.4%—15.4%] at baseline to 85.9% [82.7%—89.0%] following the health education intervention; p-value < 0.0001. The proportions of participants responding correctly to individual questions assessing belief whether SCD was the same as blood cancer, whether an individual with SCD can live to normal life expectancy and knowledge that SCD is an inherited disorder, that phenotypically normal parents may have a child with SCD in case both parents have sickle cell trait as well as knowledge that anemia, jaundice, pain, dactylitis, abdominal distension due to enlarged spleen are common signs and symptoms of SCD improved from 13.5% to 49.5% at baseline to 67.7% to 98.3% after health education intervention (Fig. 3).

### Efficacy of health education intervention on SCD

On univariate and multivariate logistic regression analysis for the efficacy of health education provided, the final model revealed the health education provided was efficacious and was associated with those who shared the received health education. It showed that, the efficacy of health education was 2.45 times higher among those who shared the received education with others than those who did not share (AOR = 2.45; 95% CI = 1.38 to 4.36) (Table 3).

**Table 3:** Regression analysis for the efficacy of health education intervention on SCD among pregnant women attending ANC in Dar Es Salaam

	**Efficacious health education**		**Univariate analysis**	***P*-value**	**Multivariate analysis**	***p*-value**
**Factors**	**Yes** **n (%)**	**No** **n (%)**	**COR [95%CI]**		**AOR [95% CI]**	
**Marital status**						
Married	339(86.7)	52 (13.3)	Reference			
Not married	67 (88.2)	9 (11.8)	1.14 [0.54–2.43]	0.73	-	-
**Level of Education**						
Illiterate/ Primary	179 (90.4)	19 (9.6)	1.99[0.89–4.45]	0.09	1.86 [0.76–4.51]	0.17
Secondary	175 (85.0)	31 (15.0)	1.19 [0.56–2.54]	0.65	1.19 [0.54–2.62]	0.67
College / university	52 (82.5)	11 (17.5)	Reference		Reference	
**Occupation**						
Employed	36 (76.6)	11 (23.4)	Reference		Reference	
Self employed	238 (87.8)	33 (12.2)	2.20 [1.02–4.75]	**0.04**	2.08 [0.99–4.63]	0.07
Housewife	132 (88.6)	17 (11.4)	2.37 [1.02–5.55]	**0.05**	2.18 [0.89–5.32]	0.09
**Mother Screened for SCD**						
Yes	198 (89.6)	23 (10.4)	1.57 [0.90–2.73]	0.1	0.2 [0.03–3.14]	0.31
No	208 (84.6)	38 (15.5)	Reference		Reference	
**If shared health education**						
Yes	278 (90.0)	31 (10.0)	2.10 [1.22–3.62]	**0.007**	2.45 [1.38–4.36]	**0.002**
No	128 (81.0)	30 (19.0)	Reference		Reference	

*COR * Crude Odd Ratio, AOR Adjusted Odd Ratio, *95%CI* Confidence Interval at 95%

### The efficacy of maternal screening on the uptake of infant screening for SCD

In assessing the efficacy of maternal screening for SCD on the uptake of infant screening, the Chi square test showed no significant difference between mothers who were screened and those who were not screened (*p*-value 0.074) (Table 4).

**Table 4:** The efficacy of maternal screening on the uptake of infant screening for SCD

Health facility	Child screened for SCD		Total	*p*-value
	**Yes**	**No**		
	**n (%)**	**n (%)**		
Sinza (Not screened)	9(3.6)	240(93.4)	249	0.074
Buguruni and Mbagala (Screened)	16(7.3)	202(92.7)	218	
Total	25	442	467	

### Predictors of infant diagnosis

In ascertaining the predictors of infant diagnosis, we performed logistic regression analysis where the final model revealed that infant diagnosis was associated with if mothers shared the received health education, occupation as well as maternal SCD status (Table 5). The odds of infant diagnosis being done were 5.39 higher in infants whose mothers shared the received education than those who did not share (AOR = 5.39; 95% CI = 1.18 to 24.67). Mothers who were employed had 6.49 higher odds of screening their infants than housewives (AOR = 6.49; 95% CI = 1.52 to 27.79). Maternal status was also seen to associate with infant diagnosis whereby those with Hemoglobin S (AS or SS) had 13.28 times higher odds of screening their infants than those who did not know their SCD status, that is, were not screened (AOR = 13.28; 95% CI = 4.11 to 42.86). There was no difference in odds of the baby being screened for SCD between mothers who had Hemoglobin AA as compared to mothers who were not screened for SCD (AOR = 1.62; 95% CI = 0.56 to 4.84).

**Table 5:** Predictors of infant diagnosis for SCD among pregnant women attending antenatal clinics in Dar es Salaam

	**Child screened for SCD**		**Univariate Analysis**		**Multivariate Analysis**	
**Maternal characteristics**	**Yes (*N* = 25)** **n (%)**	**No (*N* = 442)** **n (%)**	**COR [95% CI]**	***p*-value**	**AOR [95% CI]**	***p*-value**
**Level of education**						
Illiterate/Primary	11 (5.6)	187 (94.4)	Reference			
Secondary level	9 (4.4)	197 (95.6)	0.78 [0.31–1.92]	0.58	-	-
College /university	5 (7.9)	58 (92.1)	1.47 [0.49–4.39]	0.50	-	-
**Occupation**						
Employed	6 (12.8)	41 (87.2)	5.30 [1.43–19.70]	**0.01**	6.49[1.52–27.79]	**0.01**
Self-employed	15 (4.5)	256 (94.5)	3.12 [0.69–6.52]	0.19	1.75[0.53–5.70]	0.36
Housewife	4 (2.7)	145 (97.3)	Reference		Reference	
**Maternal Status (*n* = 467)**						
AA	8 (4.2)	182 (95.8)	1.31 [0.48–3.55]	0.60	1.62[0.56–4.84]	0.38
AS / SS	9 (29.0)	22 (71.0)	12.17[4.27–34.70]	** < 0.0001**	13.28[4.11–42.86]	** < 0.0001**
Not screened	8 (3.2)	238 (96.8)	Reference		Reference	
**Level of Knowledge post Training**						
Poor Knowledge	2 (3.0)	64 (97.0)	1.95 [0.45–8.46]	0.37	-	-
Good knowledge	23 (5.7)	378 (94.3)	Reference			
**If Shared received HE (*n* = 467)**						
Yes	23 (7.4)	286 (92.6)	6.27 [1.46–26.96]	**0.01**	5.39[1.18–24.67]	**0.03**
No	2 (1.3)	442 (94.6)	Reference		Reference	

## Discussion

Improving community knowledge on SCD is critical to enhancement of the quality of care for SCD. Particularly, empowering pregnant mothers with good knowledge on SCD has the potential to have a big impact as they are the ones mostly involved in care of the young ones, including those with SCD, from channeling them to diagnosis to day-to-day care throughout life. Further, knowing of maternal status will inform the risk of inheriting the sickle gene for the child to be born and hence influence readiness to screen the baby as well as early linkage to care, upon diagnosis. Here we have shown that a SCD health education program to pregnant women as well as maternal screening for SCD using a point-of-care diagnostic in the context of ANC in Tanzania were both feasible and efficacious, and predicted the uptake of infant diagnosis for SCD. These findings have the possibility to inform public health initiatives, particularly in areas with a high burden of SCD in sub-Saharan Africa.

Health education programs focusing on SCD among pregnant women are uncommon, representing a missed opportunity to utilize the antenatal clinics (ANC). Through a simple health education intervention, we have demonstrated an improvement in the proportion of mothers knowledgeable on SCD from below a quarter to more than three quarters. The observed efficacy of a community-based SCD health education intervention is similar to that reported in diverse populations in Saudi Arabia and Nigeria [14, 15]. However, our study is one of the first to test the efficacy of a SCD-focused education intervention among pregnant women attending ANC. The ANCs have been successfully utilized in providing health education on HIV and other conditions [16–19], and thus success in proving health education on SCD opens the possibility to consider integrating health education on SCD into the comprehensive care package for pregnant women [10], particularly in areas with high prevalence of SCD. Before health education intervention, the level of knowledge among pregnant women in our cohort was significantly predicted by the level of education, hearing of SCD at school and having lived with a patient with SCD [20]. However, following health education intervention, the efficacy of the intervention was only predicted by having shared the received information with others. On one hand, this shows that education interventions that are simple in design can efficaciously improve knowledge of pregnant women from diverse backgrounds, including those with low level of education and no prior personal experience with patients with SCD. On the other hand, our findings indicate that it may be of value to encourage participants of health education interventions to share their knowledge with others as this presumably enhances personal knowledge through active search for facts and the delivery process itself [21].

Despite the high prevalence of SCD in Tanzania (13% to 20%) [3, 4], only 2 participants knew their SCD status before intervention [20]. This was quiet similar to many other studies which showed low awareness of personal SCD status among different groups [8, 9, 12, 20]. However, maternal screening and health education interventions done in this study have raised awareness of personal SCD status where 91.6% of the participants who were screened during this study remembered their SCD status several months since screening and 1 participant from the non-screening group made personal effort to screen for SCD and remembered her status. This is similar to another study which showed that post-intervention health education resulted in the uptake of screening by 11.88% of the respondents who had a genotype test done after the health education program [22].

Following delivery, the uptake of infant diagnosis for SCD was observed to be higher among mothers who were employed, those who shared the received health education with others and those who carried the hemoglobin S gene (HbAS and SS). This study has shown that there was no difference in the uptake of infant screening among mothers who were screened and those who were not screened. This may be due to the large number of participants who were HbAA among those who were screened who might have behaved similar to those who were not screened. This is supported by the observation that mothers who were HbSS/AS were more likely to screen their infants for SCD compared to those who were HbAA or were not screened.

The observation that mothers who were carriers of hemoglobin S (HbAS and SS), but not HbAA, proceeded to screen their babies for SCD indicates that empowering mothers with the knowledge of the personal SCD status may influence behavior to test their babies for SCD. In areas where universal newborn screening is not practices but the prevalence of SCD as well as fertility rates are high [3, 4, 23], empowering women to know their personal SCD status may be a cost-effective way to increase the uptake of newborn and infant diagnosis for SCD through programmatic and personal initiatives, and is in line with recommendations for targeted screening for SCD [5, 24, 25]. The scalability of these interventions may however be hampered by limited baseline infrastructure and programs in support of both health education and screening interventions for SCD in our settings. For instance, there is no universal newborn screening services in our country, and most of the district and other lower-level health facilities have no SCD clinics. Further, neither SCD health education nor screening is being conducted at ANCs from the national to lower-level health facilities. Nonetheless, the high rates of attendance to ANC and successes of maternal health education for HIV and other diseases is a starting point towards success of SCD health education and screening interventions.

However, this study was not without limitation. We had big number of participants who were loss to follow-up due to changes in place of domicile as well as challenges in establishing communication through the phone. This was mitigated by enrolling 160 participants above the minimum required sample size to ensure that the statistical analyses remained sound. The fact that there were no newborn SCD screening services at the study sites, as is the case in most of health facilities in the country, meant that participants could not get the screening services for their babies at facilities near their physical locations. This was mitigated by directing mothers to attend to reginal referral hospitals or national hospital, whichever was near their places of domicile, for their babies to be screened for SCD.

Fathers have a major role to play during and after pregnancy as main supporters of the wives both socially, emotionally and financially. It is therefore important for fathers to receive the health information direct from the healthcare workers and get the chance to ask questions to clear doubts if any. It is even more important to involve fathers in education as well as screening of genetic conditions such as SCD, since the fathers directly predict the outcome of the condition of the baby. However, in this study, we did not involve fathers in either health education or screening due to limited resources as well as time to make follow-up in both groups.

## Conclusion

We have demonstrated that maternal health education and maternal screening for SCD are feasible and efficacious interventions in raising awareness and improving the uptake of infant diagnosis for SCD. These interventions are strongly recommended to be integrated in the comprehensive care package for pregnant women attending antenatal clinics, particularly in sub-Saharan Africa where the burden of SCD is high. This will in succession buildup the community with right information on SCD, who know their SCD status and linked to care at early stage of diagnosis which will all together reduce morbidity and mortality for newly diagnosed patients and finally the burden of SCD at large.

## Data Availability

The datasets used and/or analysed during the current study are available from the corresponding author on reasonable request.

## Abbreviations

ANC: Antenatal care
AOR: Adjusted Odd Ratio
CI: Confidence Interval
COR: Crude Odd Ratio
Hb: Hemoglobin
Hb AA: Homozygous Adult Hemoglobin
Hb AS: Heterozygous Sickle Hemoglobin
Hb SS: Homozygous Sickle Hemoglobin
HE: Health education
MUHAS: Muhimbili University of Health and Allied Sciences
NBS: New Born Screening
RCH: Reproductive and Child Health
SCD: Sickle Cell Disease
SCT: Sickle Cell Trait
SPARCO: Sickle Pan African Research Consortium
